# A New Mint1 Isoform, but Not the Conventional Mint1, Interacts with the Small GTPase Rab6

**DOI:** 10.1371/journal.pone.0064149

**Published:** 2013-05-30

**Authors:** Anika Thyrock, Edith Ossendorf, Martin Stehling, Mark Kail, Tanja Kurtz, Gottfried Pohlentz, Dieter Waschbüsch, Simone Eggert, Etienne Formstecher, Johannes Müthing, Klaus Dreisewerd, Stefan Kins, Bruno Goud, Angelika Barnekow

**Affiliations:** 1 Department of Experimental Tumorbiology, University Muenster, Muenster, Germany; 2 Max Planck Institute for Molecular Biomedicine, Muenster, Germany; 3 Institute for Hygiene, University Muenster, Muenster, Germany; 4 Hybrigenics Services, Paris, France; 5 Department of Human Biology and Human Genetics, University Kaiserslautern, Kaiserslautern, Germany; 6 Centre de Recherche, Institute Curie, Paris, France; Thomas Jefferson University, United States of America

## Abstract

Small GTPases of the Rab family are important regulators of a large variety of different cellular functions such as membrane organization and vesicle trafficking. They have been shown to play a role in several human diseases. One prominent member, Rab6, is thought to be involved in the development of Alzheimer’s Disease, the most prevalent mental disorder worldwide. Previous studies have shown that Rab6 impairs the processing of the amyloid precursor protein (APP), which is cleaved to β-amyloid in brains of patients suffering from Alzheimer’s Disease. Additionally, all three members of the Mint adaptor family are implied to participate in the amyloidogenic pathway. Here, we report the identification of a new Mint1 isoform in a yeast two-hybrid screening, Mint1 826, which lacks an eleven amino acid (aa) sequence in the conserved C-terminal region. Mint1 826, but not the conventional Mint1, interacts with Rab6 via the PTB domain. This interaction is nucleotide-dependent, Rab6-specific and influences the subcellular localization of Mint1 826. We were able to detect and sequence a corresponding proteolytic peptide derived from cellular Mint1 826 by mass spectrometry proving the absence of aa 495–505 and could show that the deletion does not influence the ability of this adaptor protein to interact with APP. Taking into account that APP interacts and co-localizes with Mint1 826 and is transported in Rab6 positive vesicles, our data suggest that Mint1 826 bridges APP to the small GTPase at distinct cellular sorting points, establishing Mint1 826 as an important player in regulation of APP trafficking and processing.

## Introduction

Cellular transport mechanisms are regulated by numerous proteins involved in signal transduction. Among these are the members of the Rab protein family, the largest group of small GTPases within the Ras superfamily [1].They are known to be involved in a variety of steps during transport processes, such as membrane docking and fusion, budding events and vesicular movement along cytoskeletal tracks [2]. One of the most widely studied Rab GTPases is Rab6, of which four isoforms have been described: Rab6A, the alternative splice variant Rab6A’, the tissue-specific form Rab6B and Rab6C, a retrogene derived from Rab6A’ [3]–[6]. As a very multifunctional protein, Rab6A is known to regulate the retrograde vesicular trafficking from the Golgi apparatus to the endoplasmatic reticulum (ER) via Bicaudal-D [7]–[10]. Rab6B is thought to fulfill this task in neuronal cells [11]. Additional functions of Rab6 include the transport of early endosomes and recycling endosomes towards the trans-Golgi network and the trafficking of exocytotic vesicles towards the plasma membrane [10], [12], [13]. Several studies have also suggested the involvement of Rab6 in various diseases such as Lowe’s Syndrome or HIV [14], [15]. There is now evidence that the small GTPase plays a role in the pathology of Alzheimer’s Disease (AD) [16]–[20].

AD is the most common neurodegenerative disorder worldwide [21]. One of the characteristic hallmarks in the pathology of AD is the presence of extracellular aggregates, consisting of amyloid-beta (Aβ) in the brains of patients [22]. These plaques derive from the proteolytical cleavage of the amyloid precursor protein (APP), a type I transmembrane protein [23]. The amyloidogenic processing is performed sequentially by β- and γ-secretases [24]–[26]. In the non-amyloidogenic pathway Aβ fragments are not produced because APP is initially cleaved inside the Aβ peptide sequence by α-secretases, followed by γ-secretase processing [27]–[29]. The way in which APP is cleaved depends on its transport route: Amyloidogenic processing is thought to take place in endosomes and lysosomes, whereas the non-amyloidogenic cleavage is performed mostly at the plasma membrane [30], [31].

There are many different proteins that influence the transport processes of the amyloid precursor protein, among them the Mint adaptor proteins, which bind to the C-terminal YENPTY motif of APP [32]. The family of Mint adaptor proteins comprises three previously described members: The neuronal Mint1 and Mint2 and the ubiquitously expressed Mint3 [33]–[35]. The three Mint proteins have a highly conserved C-terminus, which consists of one phosphotyrosine-binding (PTB) and two PDZ domains. Mint1 displays an additional Munc-interacting domain and a CASK-interacting domain [36], [37]. Mint proteins seem to be essential for survival, since Mint1/2 knockout mice die at birth or show a lower average weight and motor defects [38].

In this manuscript we report the discovery of a new Mint1 isoform, Mint1 826, which lacks an eleven amino acids sequence in the PTB domain. We show that Mint1 826 is a transcribed gene by detection of a specific mRNA sequence as well as the identification of the Mint1 826 protein from tissue samples by mass spectrometry. In contrast to the previously described Mint1, we show that it is able to interact with the active form (GTP-bound) of Rab6 via its PTB domain. Mint1 826 exhibits a different intracellular distribution in comparison to the previously described Mint1, as it clearly accumulates in the Golgi area. Our observations that the deleted sequence does not influence the ability of Mint1 826 to interact with APP and that Rab6 and APP co-localize in moving vesicular structures, supports the hypothesis that Mint1 826 might be an important adaptor for Rab6-driven APP transport.

## Materials and Methods

### Ethics

Human brain and testis samples were obtained from the Human Gene Bank (which is now part of BrainNet Europe) and S. Kliesch, Muenster, Germany respectively. For the acquisition of both samples no approval of an ethics committee was needed since it took place before 1999. Nevertheless, the patients gave written informed consent that their tissue could be used for experimental purposes post mortem as required by law. This consent was general and not restricted to specific studies, thus there was no need to contact an ethical review board for this current study.

All animals were housed, cared for, and experiments conducted in accordance with approved protocols from the University of Kaiserslautern/"Stadt Kaiserslautern-Referat Umweltschutz", project number: 15/73/10-Bu approval date: 11.01.2010.

#### Plasmids

Most of the plasmids used have been described before [18], [39]. To construct the pACT2 Mint1 826 plasmid, the corresponding sequence was isolated from the yeast two-hybrid clone pP6 Mint1 (bp1314–2307 Δ1483–1515) by PvuII cleavage and inserted into a PvuII cleaved pACT2 Mint1 vector. pGEX Mint1 826 was created by inserting the SacI fragment from pACT2 Mint1 826 into a SacI digested pGEX Mint1 vector (the respective 3′ SacI site derived from the Mint1 3′ UTR region that is present in both vectors). APP695-RFP contains a C-terminally fused mRFP (monomeric red fluorescent protein) tag and was cloned via PCR based mutagenesis in vector pcDNA3.1, as described earlier [19]. All of the other plasmids mentioned were cloned using standard molecular biological techniques.

#### Antibodies

The monoclonal antibody against GFP (JL8) was purchased from Clontech, Heidelberg, Germany and diluted 1∶4000. For Western blotting, the monoclonal antibody against Mint1 (A-12) was purchased from Santa Cruz Biotechnology, Inc., Heidelberg, Germany and diluted 1∶500. For the detection of APP in Western blot analyses an anti-APP C-term antibody distributed by Calbiochem, Merck Millipore, Darmstadt, Germany was applied 1∶5000, for detection of APP in immunofluorescence analyses it was diluted 1∶1000. Mint 1 was stained using an antibody (clone 23) purchased from Becton Dickinson, Heidelberg, Germany (1∶25). Anti-mouse IgG HRP was purchased from Cell Signaling Technology ®, Danvers, MA, USA and applied 1∶1000. For localization studies, intracellular Rab6A was stained using a monoclonal antibody (5B10, dilution: 1∶50) [40], which was directly conjugated with OY594 by Luminartis GmbH, Muenster, Germany. The polyclonal antibody against Rab6B (Institute Curie, Paris, France) was affinity purified and applied at 1∶50 dilution. GM130 was stained with an antibody distributed by Becton Dickinson, Heidelberg, Germany (clone 35, 1∶200). As secondary antibodies, anti-mouse IgG Alexa Fluor®488 (Invitrogen, Karlsruhe, Germany, 1∶1000), anti-mouse IgG Alexa Fluor®647 (Invitrogen, Karlsruhe, Germany, 1∶500) and anti-rabbit IgG Alexa Fluor®594 (Invitrogen, Karlsruhe, Germany, 1∶1000) were used.

#### Cell culture

All cell lines used for our studies were cultivated in DMEM (Biochrom AG, Berlin, Germany) supplemented with 10% fetal calf serum (Pan-Biotech GmbH, Aidenbach, Germany) and 2 mM glutamine.

CHO K1 (ATCC® number: CCL-61) [18] and HeLa T-Rex™ (Invitrogen, Karlsruhe, Germany) cells have been handled as described previously [39].

MEF dko APP695 AA12 cells were a kind gift of U. Müller, Heidelberg, Germany. In this cell line APP695 and APLP2 were knocked out and APP695 retransfected [41].

The preparation of primary neurons (isolated from mice purchased from Janvier, Saint Berthevin Cedex, France) has been described previously [42].

For localization studies of Mint1 and Mint1 826, 3T3 Swiss cells (ATCC® number: CCL-92) were transfected with pEGFP Mint1 or pEGFP Mint1 826 respectively using the TurboFect™ transfection reagent as described in the manufacturer’s manual (Thermo Fisher Scientific, St. Leon-Rot, Germany). Cells were fixed 24 h after transfection.

#### Flow cytometry based FRET analyses

Flow cytometry based FRET analyses have been performed as described previously [39].

#### Yeast two-hybrid analyses

The reporter strain Y190 (Clontech, Heidelberg, Germany) was co-transformed and colonies were analyzed as described previously [8], [43].

For the initial Rab6B Q72L screen, the coding sequence of Rab6B Q72L was cloned into pLex9 as a C-terminal fusion to LexA. The construct was used as bait to screen at saturation a highly complex, random-primed human placenta cDNA library constructed into the plasmid pP6. pLex9 and pP6 derive from the original pBTM116 [44] and pGADGH [45] plasmids, respectively. More than 130 million clones (13-fold the complexity of the library) were screened, using a mating with the Y187 (MATα) and L40ΔGal4 (MATa) yeast strains as previously described [46]. Positive colonies were selected on a medium lacking tryptophan, leucine and histidine supplemented with 5 mM 3-aminotriazole. The prey fragments of the positive clones were amplified by PCR and sequenced at their 5′ and 3′ junctions. The resulting sequences were used to identify the corresponding interacting proteins in the GenBank database (NCBI) using a fully automated procedure.

#### Preparation of mouse brain lysate

Frozen mouse brains were pestled in liquid nitrogen. The cells were lyzed in immunoprecipitation (IP) buffer (25 mM Tris pH 8.0, 50 mM NaCl, 0.5% Triton X-100, Complete EDTA free (Roche Diagnostics GmbH, Mannheim, Germany)) with a Potter S homogenizer (10 strokes at 1000 rpm) [47]. Finally, the lysate was incubated on ice for 30 min and cleared by centrifugation at 15000×g for 1 h at 4°C.

#### GST pulldown experiments

The expression and preparation of GST fusion proteins has been carried out as described before except that an incubation temperature of 37°C was used instead of 30°C [48]. For the *in vitro* GST binding assay 10 µg of the GST Rab6A Q72L fusion protein were bound to 10 µl of glutathione-Sepharose™ 4B beads (GE Healthcare, Freiburg, Germany) for 1 h at 4°C in PBS with 1% Triton X-100 and Complete EDTA free adjusted to 300 µl. Beads were then washed with PBS/Triton X-100 for three times and incubated overnight at 4°C with 1 mg of mouse brain lysate, which had been cleared with GST-coupled glutathione Sepharose™ 4B beads for 3 h at 4°C. After the overnight incubation, beads were washed three times with IP buffer and bound proteins were eluted from the beads with sample buffer. Samples were analyzed by SDS-PAGE (7%) and Western blotting [49].

Other GST pulldown experiments using GST Mint1/Mint1 826 PTB and lysate from stably transfected HeLa T-REx™ cell lines, GST Rab6 and purified Mint1 826 as well as GST Mint1/Mint1 826 PTB and MEF lysate were performed accordingly except that pulldown buffer (10 mM Tris pH 7.4, 150 mM NaCl, 1 mM MgCl_2_, 1 mM CaCl_2_, 0.2% Triton X-100, Complete EDTA free) was used instead of IP buffer. Changes in lysate concentrations and incubation times are stated in the figure legends.

Thrombin cleavage was performed according to the manufacturer’s manual (GE Healthcare, Freiburg, Germany).

#### Immunocytochemistry

To prepare the cells for fluorescence analyses, culture dishes were washed three times with PBS and fixed on ice for 15 min using 4% paraformaldehyde in 250 mM Hepes (pH 7.4). All following steps were performed at room temperature. Cells were incubated in 8% pre-chilled paraformaldehyde in 250 mM Hepes (pH 7.4) for 30 min and washed three times in PBS. After that the coverslips were quenched for 10 min with 50 mM NH_4_Cl and after another washing procedure permeabilized in 0.2% Triton X-100 in PBS for 5 min. Cells were then washed in a PBS solution containing 0.2% gelatine. Blocking was performed for 30 min in a PBS/0.2% gelatine solution containing 10% goat serum (University Giessen, Germany). The primary antibody was diluted in the PBS/0.2% gelatine solution with 2% goat serum. After a 30 min incubation in the antibody solution the coverslips were washed three times with PBS/0.2% gelatine and incubated with the secondary antibody for 15 min. Finally, the coverslips were washed with PBS/0.2% gelatine, PBS and distilled water three times each and mounted with 8 µl of Mowiol 4–88/DABCO (Hoechst, Frankfurt a.M., Germany).

Quantitative co-localization studies between endogenous Rab6A and EGFP Mint1 or EGFP Mint1 826 respectively were performed using the cell∧F software from Olympus, Hamburg, Germany. First of all, two different regions of interest (ROI) were defined: One containing the complete cell, the other one containing the Rab6A staining at the Golgi apparatus. Nonspecific background staining was substracted by applying the “Background Substraction” tool using another ROI outside the photographed cells. Finally, the median gray scales of the complete cell and the Golgi apparatus were measured by using the option “Measure”, “ROI”, “Average Gray Value”. For comparison of the amount of Mint1 located in the Golgi area, the ratio of the average gray value at the Golgi area to the average gray value of the complete cell was determined.

For co-localization studies between Mint1 826, APP and GM130, HeLa cells were plated on glass coverslips (Marienfeld, Lauda Koenigshofen, Germany) at a density of 35.000 cells/well in a 24 well plate one day before transfection. 2–3 hours before transfection, the media was changed to 500 µl fresh HeLa culture media (DMEM +10% FBS (HyClone, Thermo Fisher Scientific, St. Leon-Rot, Germany)+Penicillin/Streptomycin (Sigma-Aldrich Chemie GmbH, Taufkirchen, Germany) +1% 200 mM L-Glutamin) per well. 1 µg DNA (for co-transfections: 2×0,5 µg DNA) was mixed with 87 µl of a 10 mM Tris/HCl pH 7,5 solution and 12,4 µl of a 2 M CaCl_2_ solution. The mixture was added to 100 µl 2×HBS (280 mM NaCl, 1,5 mM Na_2_HPO_4_, 50 mM HEPES (at a final pH of 7,12–7,13)) under aeration conditions. Afterwards, the solution was added dropwise to one well. After 3 hours incubation at 37°C and 5% CO_2_, a glycerol shock (15% glycerol in 1×HBS) was performed for 2 minutes at room temperature. Subsequent two washing steps with plain DMEM media followed. Then normal HeLa culture media was added. Cells were fixed 18–20 hours after transfection in 4% paraformaldehyde/4% sucrose in phosphate buffer solution for 10 minutes at 37°C. The cells were permeabilized for 10 minutes at room temperature in 0,1% NP40 in PBS and blocked for 1 hour in 5% goat serum in PBS. The primary antibody GM130 (cis-Golgi marker, BD-Bioscience) was added 1∶200 overnight in 1% goat serum in PBS at 4°C. The following day, the secondary antibody Alexa-Flour 647 (Molecular Probes, Invitrogen, Karlsruhe, Germany) was added 1∶500 in PBS and 1% goat serum for one hour at room temperature and cells were embedded in Mowiol. GFP Mint1 826 was visualized via reflector 488 and APP-RFP via reflector 568. Z-stack imaging was performed with the fluorescence microscope Axio observer Z.1 from Zeiss with the software Axiovision 4.8.1 and analysis followed via ImageJ. Co-localization studies between Mint1 826, APP and GM130 have been performed in primary mouse neurons, additionally. Therefore, the cells have been isolated in stage E 14. Transfection of the neurons (seven days *in vitro* (DIV7)) has been described previously [50].

#### Live cell imaging

For live cell imaging, CV1 cells (ATTC® number: CCL-70) were seeded onto IBIDI 8 well chambers and transfected with Lipofectamine 2000™ according to the manufacturer’s manual. 18 hours after transfection cells were analyzed with a Zeiss LSM5 live inverted microscope at 37°C. All images were taken in the LSM mode as 8 bit images using a two-track recording setup. Green and red channels were recorded sequentially for each time point. Laser power, pinhole and detector gain were adjusted as needed.

#### Detection of Mint1/Mint1 826 mRNA

mRNA was isolated from total RNA (derived from human brain or testis tissue) using the PolyATtract® mRNA Isolation System II from Promega, Mannheim, Germany according to the manufacturer’s manual. This mRNA was used as template for RT PCR (applied kit: First Strand cDNA Synthesis Kit for RT-PCR, Roche Diagnostics GmbH, Mannheim, Germany). The resulting cDNA was tested for the Mint1 826 sequence as described below. The area containing the deletion was amplified using the primers: 5′- ATCCATGGATTCATTCCCAACCTACGTTG-3′and 5′-CTGCTCGAGAGATCTTCGGGGTTAATCC-3′. Mint1 826 was detected by applying a specific primer, which recognizes Mint1 826 but not the conventional Mint1 by binding at the 3′ end of exon 5 and the 5′ end of exon 7. Primer sequences: 5′-AGCAGGATCAAGGCTCCTG-3′ and 5′-CTGCTCGAGAGATCTTCGGGGTTAATCC-3′. Samples were analyzed using agarose gel electrophoresis (3% NuSieve GTG, Takara, Clontech, Heidelberg, Germany) and verified by sequencing.

The conventional Mint1 was detected by using the primers 5′-ATCCATGGATTCATTCCCAACCTACGTTG-3′and 5′-CTGCTCGAGAGATCTTCGGGGTTAATCC-3′. Again, samples were analyzed using gel electrophoresis and verified by sequencing.

cDNA libraries were purchased from BD Biosciences, Clontech, Heidelberg, Germany.

#### Sample isolation for mass spectrometry

Mint1/Mint1 826 were immunoprecipitated from 2.5 mg mouse brain lysate using 2 µg of the A-12 antibody as well as 10 µl Protein G Sepharose beads (GE Healthcare, Freiburg, Germany) and separated by SDS-PAGE (7%). Several samples were excised from the gel and extracted in PBS/0.1% SDS +28 µg/ml aprotinin (AppliChem, Darmstadt, Germany) at 37°C overnight. Dissolved protein was desalted using Roti®Spin centrifugation tubes (30 kDa cut off, Carl-Roth GmbH, Karlsruhe, Germany) and concentrated by vacuum centrifugation. Subsequently, samples were separated by SDS-PAGE.

#### In-gel digest

Coomassie-stained protein bands were excised and cut into smaller pieces. Subsequently, 500 µl of pure acetonitrile (ACN, Merck, Darmstadt, Germany) was added and the mixture was shaken until the gel became white and shrank. The supernatant was removed and the gel pieces were dried *in vacuo*. The dried gel pieces were allowed to soak thermolysin (Sigma-Aldrich Chemie GmbH, Taufkirchen, Germany) solution (0.25 µg/µl) at ambient temperature for 30 min. The excess of protease solution was removed and the gel pieces were covered with 25 mM ammonium hydrogen carbonate and incubated over night at 65°C. Proteolytic peptides were subsequently extracted with 250 µl of 25 mM ammonium hydrogen carbonate (Fluka, Buchs, Switzerland), 50% ACN/2.5% formic acid (FA, Merck, Darmstadt, Germany), 80% ACN/2.5% FA, and pure ACN. The combined extracts were dried *in vacuo*.

#### ZipTip C_18_-desalting

For desalting of in-gel digested proteins ZipTip pipette tips (Millipore, Billerica, USA) were equilibrated three times with 10 µl of 50% ACN and five times with 10 µl of 0.1% Trifluoroacetic acid (TFA, Carl-Roth GmbH, Karlsruhe, Germany). The proteolytic peptides were dissolved in 10 µl of 0.5% TFA and loaded onto the tips. After washing three times with 10 µl of 0.1% TFA the peptides were eluted five times with 10 µl of 50% ACN/0.1% TFA, five times with 10 µl of 50% ACN/0.1% TFA, and three times with 10 µl of pure ACN. The combined eluates were dried *in vacuo* and redissolved in 10 µl of 40% methanol/0.5% FA (Merck, Darmstadt, Germany) for mass spectrometric analysis.

#### Mass spectrometry (MS)

The proteolytic peptides derived from in-solution digests were analyzed by nano electrospray ionization (nanoESI). Performing MS/MS experiments on peptide ions allowed for deducing their amino acid sequences from fragment ion spectra. NanoESI MS experiments were carried out by use of a SYNAPT G2-S mass spectrometer (Waters, Manchester, UK) equipped with a Z-spray source in the positive ion sensitivity mode. Typical source parameters were: source temperature: 80°C, capillary voltage: 0.8 kV, sampling cone voltage: 20 V, and source offset voltage: 50 V. For low energy collision induced dissociation (CID) experiments, the peptide precursor ions were selected in the quadrupole analyser, subjected to ion mobility separation (IMS; wave velocity 850 m/s, wave height 40 V, nitrogen gas flow rate 90 ml/min, and helium gas flow rate 180 ml/min), and fragmented in the transfer cell using a collision gas (Ar) flow rate of 2.0 ml/min and collision energies up to 100 eV (*E*_lab_).

## Results

### A Novel Mint1 Isoform, Mint1 826, Interacts with Rab6

To search for new interacting partners of Rab6B, a yeast two-hybrid (YTH) screen was performed using Rab6B Q72L as the bait protein and a human placenta cDNA library as prey. A clone was isolated that contained a fragment, which was identified as Mint1 (pP6 Mint1, aa 438–769) by partially sequencing from both ends. Besides the tissue-specific Rab6B, also the ubiquitously expressed Rab6A and Rab6A’ displayed a significant interaction signal with pP6 Mint1 (Table 1). On the other hand, pP6 Mint1 did not interact with a variety of other Rab GTPases including RhoA, H-Ras and Ypt6, the yeast homologue of Rab6 (Table 1).

**Table 1:** Rab specificity of the interaction with Mint1.

prey plasmid	bait plasmid	his3	β -gal
pP6 Mint1 (aa 438–769)	pAS Rab6A Q72L	+++	+++
	pAS Rab6A‘ Q72L	+++	+++
	pAS Rab6B Q72L	+++	+++
	pAS Rab1A wt	−	−
	pAS Rab1B wt	−	−
	pAS Rab1B Q67L	−	−
	pAS Rab2 wt	−	−
	pAS Rab2 Q65L	−	−
	pAS Rab3A wtΔC	−	−
	pAS Rab11A wt	−	−
	pAS Rab33B wt	−	−
	pAS Rab33B Q92L	−	−
	pAS Rab33B T47N	−	−
	pAS RhoA wt	−	−
	pAS H-Ras wt ΔC	−	−
	pAS Ypt6p wt	−	−
	pAS Ypt6p Q69L	−	−
	pAS Ypt1 wt	−	−

After co-transformation, Y190 strains were cultivated in synthetic media lacking leucine, tryptophan and histidine, supplemented with 30 mM 3 AT (his3). β-galactosidase reporter gene activity was determined on replica filters using X-gal as substrate (β -gal).

– no growth on selection media or staining in β-galactosidase filter assay,+++very strong growth on selection media or staining in β-galactosidase filter assay, ΔC: without prenylation site.

**Figure 1:**
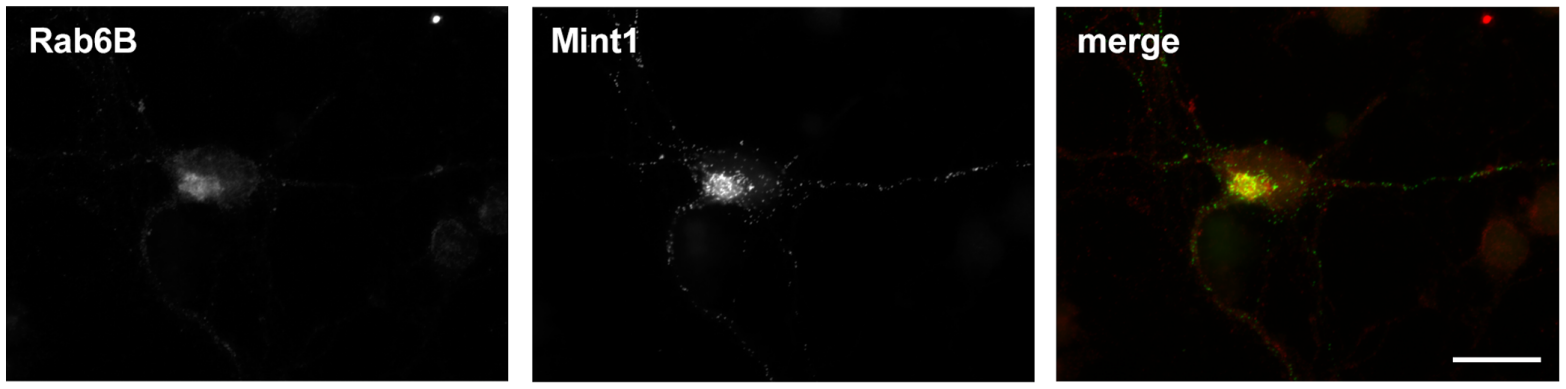
Co-localization studies with Rab6 and Mint1. Co-staining of Mint1 and Rab6B in primary mouse neurons. Cells were treated with anti-Mint1 and anti-mouse Alexa488 as well as anti-Rab6B and anti-rabbit Alexa594 antibodies. Scale bar: 20 µm.

Next, we investigated the cellular expression pattern of Mint1 and compared it with Rab6. Since Mint1 has been described as neuron specific, we chose primary mouse neurons as model and co-stained endogenous Mint1 with the neuronal Rab6 isoform Rab6B (Figure 1). A clear partial co-localization of Mint1 (which we later found out was not the previously described form of Mint1) and Rab6B was observed in the Golgi area.

Subsequently, we tested, whether the full length Mint1 protein was able to interact with Rab6 in the YTH system. The cDNA for the respective expression construct, encoding the entire Mint1 sequence (NM_001163.3), was obtained from J.P. Borg (Marseille University, France). To our surprise, the full length Mint1 did not interact with either Rab6A or Rab6B (Table 2). Since the Rab6 positive Mint1 clone that was isolated from the YTH screen was only partially sequenced, we sequenced the whole construct to explain the discrepancy. Interestingly, the YTH clone differed from the so far known Mint1 sequence by the absence of exon 6 (bp, 1483–1515, representing aa 495–505), suggesting that the deletion of this exon enables the interaction of Mint1 with Rab6. In further experiments, we constructed a full length Mint1 with the corresponding deletion and called the new Mint1 variant Mint1 826 (in comparison to the 837-aa-long conventional Mint1). In yeast co-transformation experiments, Mint1 826 showed a strong interaction with the constitutively active form of Rab6A (Table 3). In addition, we were able to show that the neuron specific Rab6 isoform Rab6B is also able to interact with Mint1 826, which is especially remarkable, considering that Mint1 is also neuron specific. A positive β-galactosidase signal was not detected with Mint1 826 and the inactive Rab6 mutants (Table 3), proving that the interaction of Mint1 826 is nucleotide-dependent. These results support the hypothesis that the deletion of the 11 amino acid sequence in Mint1 826 is responsible for the adaptor protein’s ability to bind Rab6.

**Table 2:** Full length Mint1 does not interact with Rab6.

prey plasmid	bait plasmid	his3	β-gal
pACT Mint1	pAS Rab6A Q72L	−	−
	pAS Rab6A T27N	−	−
	pAS Rab6B Q72L	−	−
	pAS Rab6B T27N	−	−
	pAS 2–1	−	−

After co-transformation, Y190 strains were cultivated in synthetic media lacking leucine, tryptophan and histidine, supplemented with 30 mM 3 AT (his3). β-galactosidase reporter gene activity was determined on replica filters using X-gal as substrate (β-gal). Mint1 (NM_001163.3) was tested against the constitutively active or inactive variant of either Rab6A or Rab6B.

– no growth on selection media or staining in β-galactosidase filter assay.

**Table 3:** Mint1 826 interacts with GTP-bound Rab6.

prey plasmid	bait plasmid	his3	β-gal
pACT Mint1 826	pAS Rab6A Q72L	++	+++
	pAS Rab6A T27N	−	−
	pAS Rab6B Q72L	+++	+++
	pAS Rab6B T27N	−	−
	pAS 2–1	−	−

After co-transformation, Y190 strains were cultivated in synthetic media lacking leucine, tryptophan and histidine, supplemented with 30 mM 3 AT (his3). β-galactosidase reporter gene activity was determined on replica filters using X-gal as substrate (β-gal). Mint1 826, which lacks aa 495–505 in comparison to the conventional Mint1, was tested against the constitutively active or inactive variant of either Rab6A or Rab6B.

– no growth on selection media or staining in β-galactosidase filter assay,++strong growth on selection media or staining in in β-galactosidase filter assay,+++very strong growth on selection media or staining in β-galactosidase filter assay.

**Figure 2:**
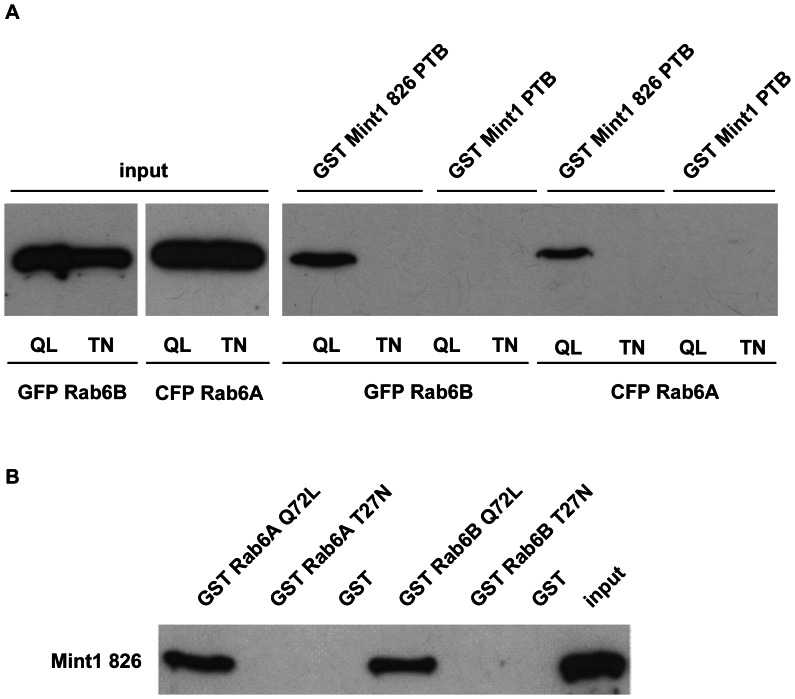
Verification of the interaction between Mint1 826 and Rab6 isoforms using GST pulldown experiments. 10 µl of Glutathione Sepharose™ 4B beads were coated with GST or the denoted GST fusion protein and then incubated with the designated prey protein. Samples were analyzed by Western blotting using an anti-GFP (a) or anti-Mint1 (b) antibody. QL: constitutively active variant (Q72L), TN: inactive variant (T27N). **A)** 5 µg of the GST Mint 1 fusion proteins were incubated with lysates of stably transfected HeLa T-REx™ cells overexpressing Rab6 GFP or CFP fusion proteins (20×input) for 1.5 h at 4°C. **B)** 1 µg of the GST Rab6 fusion protein was incubated with 300 ng of Mint 826 for 1 h at 4°C. Mint1 826 was isolated from thrombin cleaved bacterially expressed GST Mint1 826. input: 50 ng Mint1 826.

In the next step, we mapped the binding region of Mint1 interacting with the small GTPase by using truncated mutants. Previous studies have shown that the PTB domain of Mint3 is able to interact with Rab6A [18] and Rab6B (data not shown). To test, whether this applies to the new Mint1 variant, we generated a clone that contained the PTB domain only (Mint1 826 PTB, aa 438–625 Δ 495–505). In YTH experiments, Mint1 826 PTB was able to interact with Rab6A/B Q72L and Rab6A/B wildtype, but not with the inactive mutants, indicating that the PTB domain is the region of interaction for both Rab6 isoforms (Table 4). As expected, the PTB domain of the conventional Mint1 (Mint1 PTB) showed no interaction with Rab6 (Table 4). We could confirm the above results by GST pulldown experiments (Figure 2A and 2B). Thus the deletion of the 11 aa sequence in Mint1 826 enables the protein to interact with Rab6 and that the area of the interaction is the PTB domain.

**Table 4:** Mapping of the Mint1 826 interacting domain.

prey plasmid	bait plasmid	his3	β-gal
pACT Mint1 826 PTB (aa 438–625Δ495–505)	pAS Rab6A wt	++	++
	pAS Rab6A Q72L	++	+++
	pAS Rab6A T27N	−	−
	pAS Rab6B wt	++	++
	pAS Rab6B Q72L	++	+++
	pAS Rab6B T27N	−	−
	pAS 2–1	−	−
pACT Mint1 PTB (aa 438–625)	pAS Rab6A wt	−	−
	pAS Rab6A Q72L	−	−
	pAS Rab6A T27N	−	−
	pAS Rab6B wt	−	−
	pAS Rab6B Q72L	−	−
	pAS Rab6B T27N	−	−
	pAS 2–1	−	−

After co-transformation, Y190 strains were cultivated in synthetic media lacking leucine, tryptophan and histidine, supplemented with 30 mM 3 AT (his3). β-galactosidase reporter gene activity was determined on replica filters using X-gal as substrate (β-gal). Mint1 (NM_001163.3) PTB was tested against the wildtype or the constitutively active or inactive variant of either Rab6A or Rab6B. Mint1 826 PTB, which lacks aa 495–505 in comparison to the conventional Mint1 was as well tested against the wildtype or the constitutively active or inactive variant of either Rab6A or Rab6B.

– no growth on selection media or staining in β-galactosidase filter assay,++strong growth on selection media or staining in in β-galactosidase filter assay,+++very strong growth on selection media or staining in β-galactosidase filter assay.

**Figure 3:**
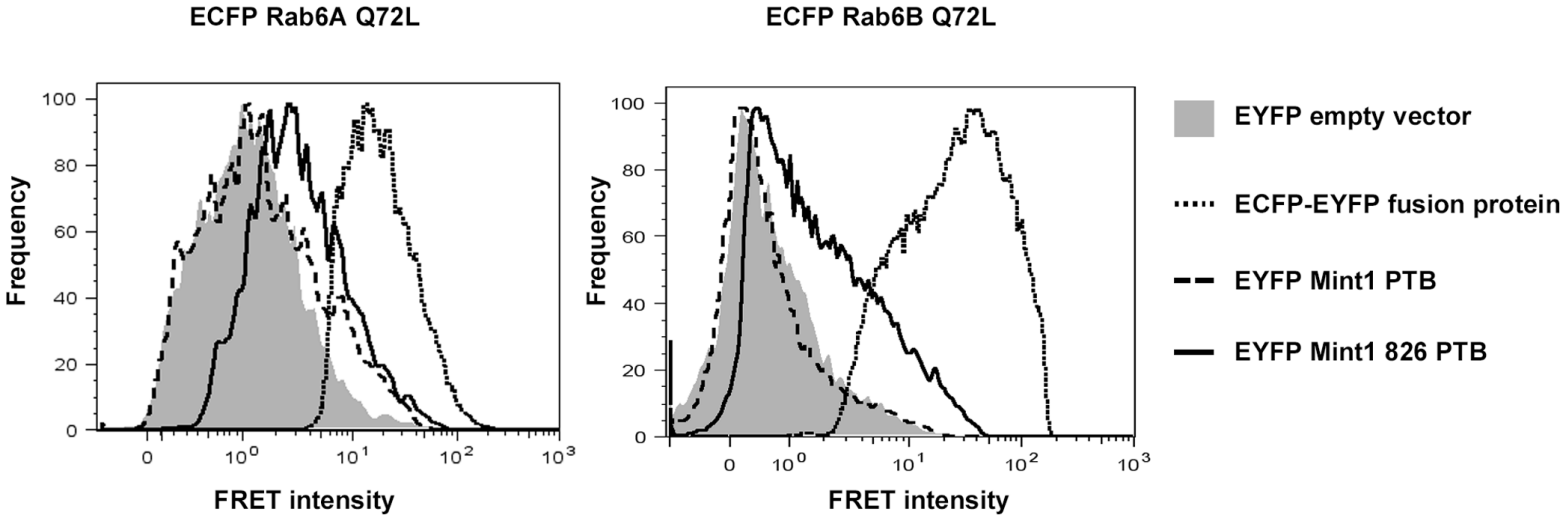
Flow cytometry based FRET analyses. HeLa T-REx cells were co-transfected with the indicated plasmids. Cells transfected with ECFP Rab6 and EYFP-C1 empty vector served as negative, EYFP–ECFP fusion protein as positive control. The graph displays EYFP fluorescence (550 nm) from 405 nm excitation of ECFP- and EYFP-double positive cells after correction of spectral bleeding of ECFP into the FRET (550 nm) channel and direct excitation of EYFP by the 405 nm laser line.

Although YTH *in vivo* experiments and *in vitro* studies like GST pulldown analyses are potential tools for detecting protein-protein interactions, a confirmation of the interplay in living mammalian cells is advantageous. One attractive method to prove that proteins interact in living cells is to use flow cytometry based FRET analysis. As additional protein components or adaptor proteins would lead to a higher distance between the CFP and the YFP fluorophor and therefore inhibit the energy transfer, a FRET signal also indicates a direct interaction between the tested proteins. As shown in Figure 3, a FRET signal could be detected in cells that were co-transfected with pECFP Rab6A/B Q72L and pEYFP Mint1 826 PTB, but not in cells expressing ECFP Rab6A/B Q72L and EYFP Mint1 PTB (Figure 3). This demonstrates that Rab6 and Mint1 826 specifically interact *in vivo*.

The results presented so far indicate that the newly discovered Mint1 variant, lacking exon 6, is able to interact with Rab6A and Rab6B via its PTB domain, whereas the previously described Mint1 protein appears not to be an interacting partner. Biochemical experiments as well as studies in living human cells showed that the interaction is direct, nucleotide-dependent and Rab6-specific.

### Mint1 826 is Expressed Endogenously on mRNA and Protein Level

**Figure 4:**
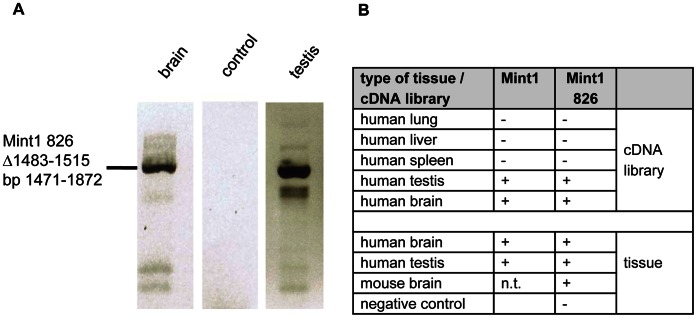
Detection of Mint1 826 mRNA. Various types of cDNA libraries derived from lung, liver, spleen, testis and brain were analyzed for the existence of Mint1 826. For this purpose a Mint1 826 specific primer that does not amplify the conventional Mint1 (tested using a linearized pACT Mint1 vector as a template, negative control) was applied in PCR analyses containing the libraries as templates. Positive results were confirmed utilizing mRNA isolated from the respective tissues. Additionally, the respective samples were also tested for the existence of the conventional Mint1 mRNA. **A)** Mint1 826-specific PCR analyses using RNA from human brain and human testis tissues. **B)** Summary of the Mint1/Mint1 826-specific PCR analyses.

Due to the lack of an antibody able to discriminate between Mint1 826 and Mint1, finding evidence for Mint1 826 via standard Western blot analyses was not possible. We therefore set out to show that Mint1 826 is a transcribed gene by detection of the specific mRNA sequence. During a search of the EST database we identified an EST clone (BE937843.1) that corresponded to the Mint1 826 sequence. We then searched for evidence of Mint1 826 expression in different types of tissue and cell lines. We designed a Mint1 826 specific primer (see Materials and Methods), which did not recognize the conventional Mint1 and used it to amplify a 401 bp fragment of Mint1 826 (Figure 4A). We first analyzed cDNA libraries representing different tissue types (Figure 4). Interestingly, Mint1 826 displayed a tissue-specific transcription pattern. Apart from some minor signals resulting from non-specific primer binding, a clear Mint1 826 signal was detected in probes of human testis and brain but not in lung, liver or spleen. To extend these results, we isolated total RNA from human brain and testis tissue as well as from murine brain, extracted mRNA from the samples and performed RT-PCRs. The cDNA was tested using the Mint1 826 specific primer. A distinct signal was detected in murine and human brain as well as in human testis samples. Together these data indicate that the endogenous human and murine Mint1 826 is predominantly transcribed in brain and testis (Figure 4). Additionally, we tested the mentioned libraries and tissue samples for the existence of the conventional Mint1. Surprisingly, it was found in all samples that also contained Mint1 826, although it has been described as being transcribed and expressed neuron-specific [33] (Figure 4B). The fact that the conventional Mint1 was found in all samples that also contained Mint1 826, strengthens the fact that Mint1 826 is not a product of accidental exon-skipping processes, neither in tissues nor in cell cultures.

**Figure 5:**
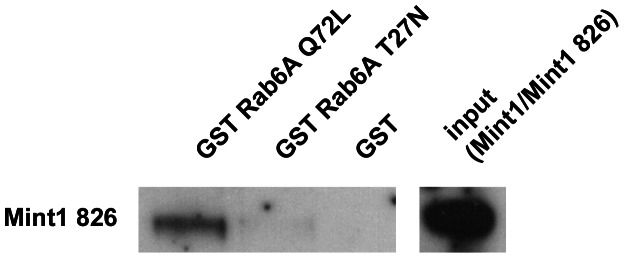
Detection of the Mint1 826 protein. For detection of the Mint1 826 protein GST pulldown assays were performed. 10 µl of Glutathione Sepharose™ 4B beads were coated with 10 µg of GST or the denoted GST fusion protein and then incubated with 1 mg of mouse brain lysate for 3 h at 4°C. Since Mint1 826 seems to exhibit a relatively low expression level, three samples were pooled for Western blot analyses. input: 15 µg mouse brain lysate.

While the presence of an mRNA transcript is strong evidence for the existence of a specific protein, it is not an ultimate proof. We thus performed GST pulldown experiments with GST Rab6A Q72L to isolate endogenous Mint1 826 from mouse brain. An immunoreactive band was detected in the GST Rab6A Q72L sample, but not in lysates incubated with GST Rab6A T27N or GST alone (Figure 5). The blotting membrane was incubated with a Mint1 specific antibody, which recognizes the N-terminus of the protein and is therefore able to recognize both Mint1 variants. Since we showed that only Mint1 826 but not Mint1 can bind to Rab6 using a variety of methods, this pulldown experiment strongly indicates that not only the transcript, but also the Mint1 826 protein is present in mouse brain tissue and that this endogenous Mint1 826 can interact with Rab6.

**Figure 6:**
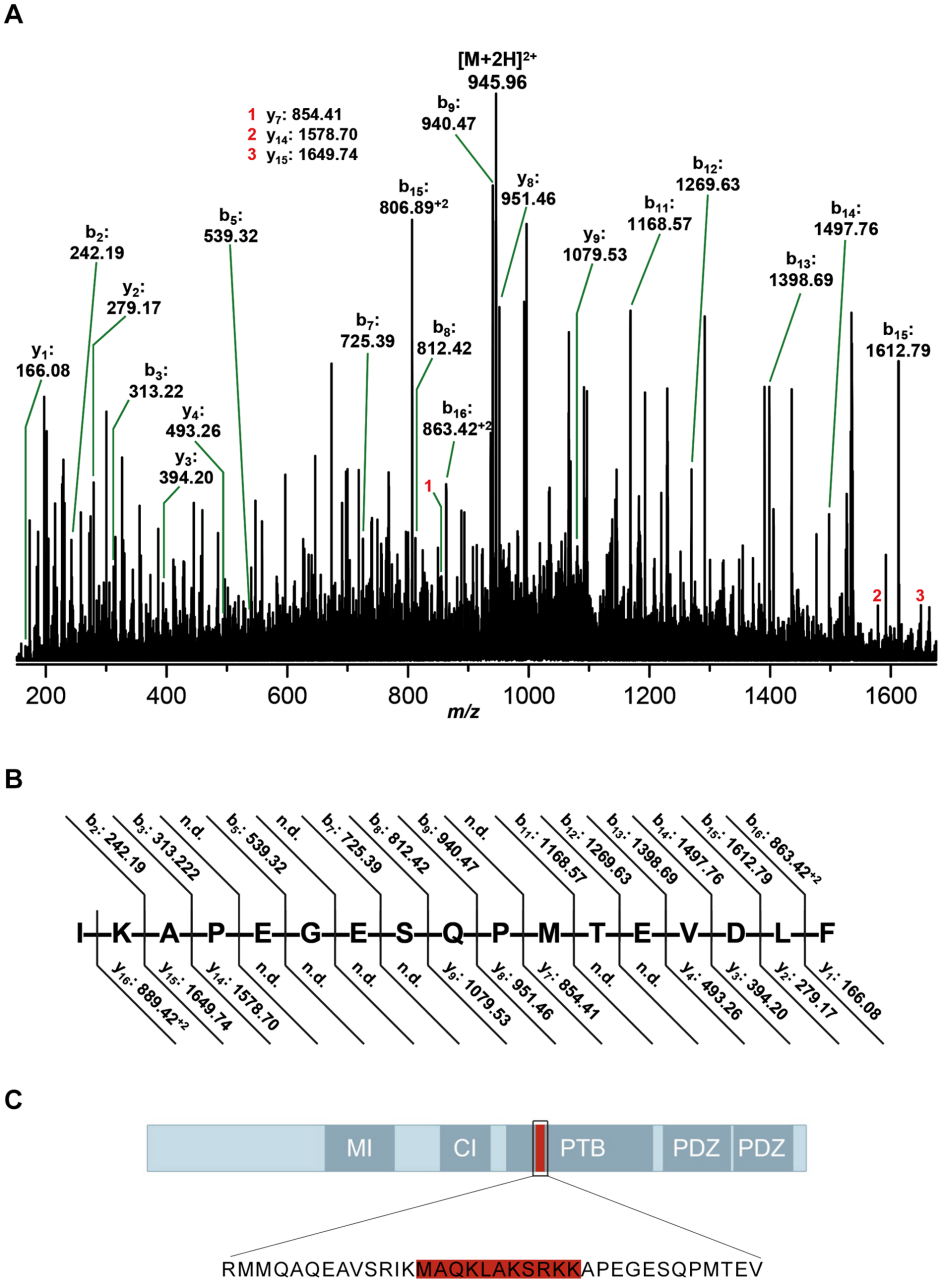
Detection of Mint 826 by mass spectrometry. **A)** NanoESI fragment ion spectrum obtained from a CID experiment on the ion mobility-separated doubly charged peptide precursor ions at m/z 945.96 derived from an in-gel proteolytic digest by thermolysin of immuno-precipitated Mint1 826 protein. **B)** Corresponding fragmentation scheme. **C)** Schematic illustration of both Mint1 isoforms. Highlighted in red: 11 aa sequence deleted in Mint1 826.

To confirm these findings we additionally analyzed Mint1 826 from mouse brain by mass spectrometry (Figure 6). Immunoprecipitated Mint1 protein (representing Mint1 as well as Mint1 826) was separated by SDS PAGE and the corresponding band was excised and subjected to in-gel digest by thermolysin as described in the Materials and Methods section. The resulting peptides were analyzed by nanoESI MS. Direct fragmentation of the doubly charged candidate precursor ions at *m/z* 945.96 in the trap cell of the hybrid mass spectrometer did not lead to clear-cut fragment ion spectra, possibly because of co-extracted compounds of similar *m/z* values. Therefore, the selected precursor ions were first separated by means of ion mobility and subsequently fragmented in the transfer cell of the instrument. Due to the extremely low abundance of Mint1 826, the CID spectrum still contained a relatively high fragment ion background. However, the clearly detectable almost complete series of b type ions, amended by a number of complementary y type ions (depicted in Figure 6A and B) unambiguously prove the presence of the peptide IK APEGESQPMT EVDLF, which lacks the 11 aa sequence found in the conventional Mint1 (Figure 6C).

### Functional Analyses of the Interaction between Mint1 826 and Rab6

We next tested, whether Mint1 826 can interact with APP and whether it co-localizes with Rab6 and APP on the subcellular level.

**Figure 7:**
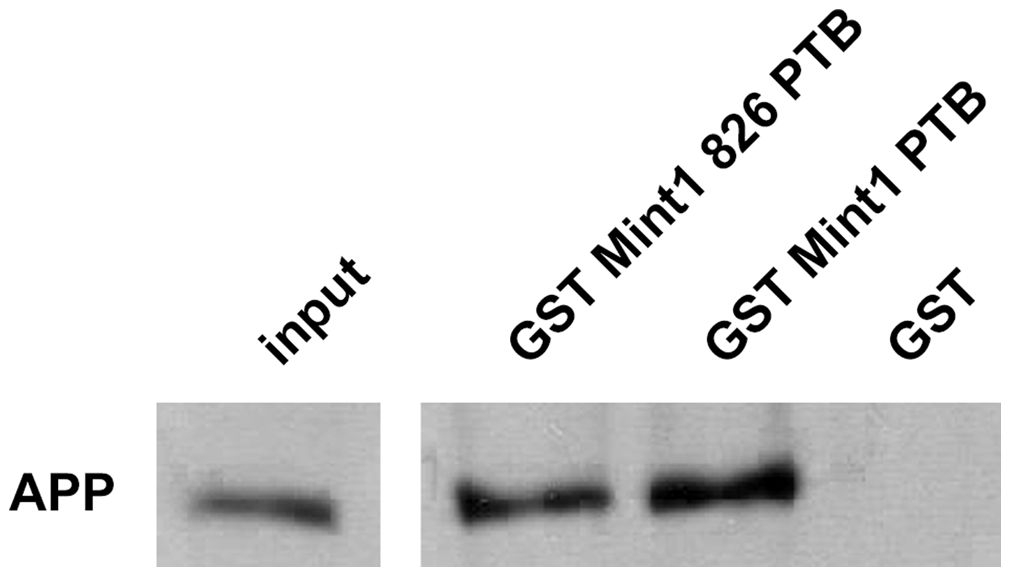
Verification of the interaction between Mint1 826 and APP. 10 µl of Glutathione Sepharose™ 4B beads were coated with 5 µg of GST or the denoted GST Mint1 fusion proteins and then incubated with MEF dko APP695 AA12 cell lysates (20 x input) for 1.5 h at 4°C. Samples were analyzed by Western blotting using an anti-APP C-terminus antibody.

The interaction of Mint1 with APP via the PTB domain is well documented [32]. To show that Mint1 826 is also capable to bind APP despite the lack of the 11 aa sequence, we performed appropriate GST pulldown experiments. GST fusion proteins of truncated mutants of Mint1 826 (Mint1 826 PTB) as well as Mint1 (Mint1 PTB) were able to bind APP695 from MEF dKO APP695 cell lines (Figure 7). These results were confirmed using GST Mint1 826 PTB/GST Mint1 PTB and lysate from CHO cells, which were transfected with pEGFP APP695 and lysed 48 h after transfection (data not shown). Additionally, we examined, whether GST Rab6 could bind APP directly, but it turned out that neither GST Rab6A nor GST Rab6B was able to associate with the amyloid precursor protein (data not shown).

**Figure 8:**
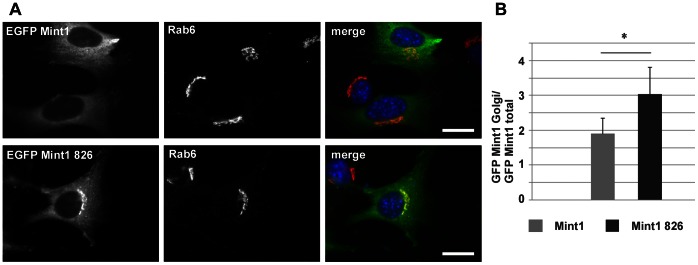
Intracellular distribution of Mint1 826 and Mint1. 3T3 Swiss cells were transiently transfected with either pEGFP Mint1 826 or pEGFP Mint1. 24 h after transfection cells were fixed and co-stained with a Rab6A specific antibody (5B10), which was conjugated with OY594 directly. **A)** Co-localization studies of endogenous Rab6A with EGFP Mint1 826 or EGFP Mint1, respectively. **B)** Quantitative analyses of the cellular distribution of Mint1/Mint1 826. Cells from two independent experiments were evaluated with the Cell∧F software (Olympus) by calculating the ratio between the average gray value of Mint1 at the Golgi apparatus and the average gray value of Mint1 in the total cell. Statistical significance was tested via student’s t-test, n = 80.

**Figure 9:**
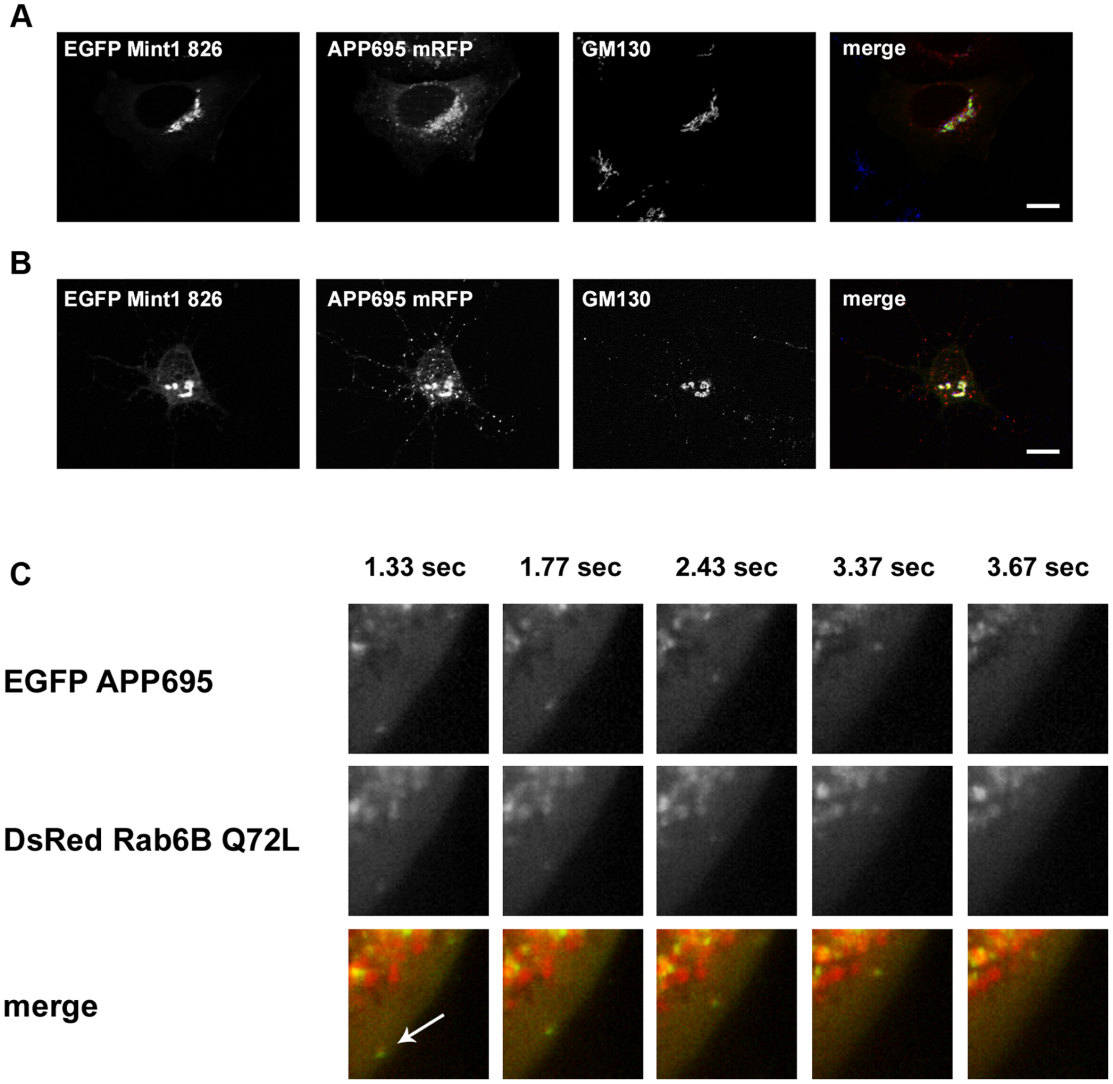
Fluorescence analyses of Rab6, Mint1 826 and APP. **A)** Co-staining of Mint1 826, APP and GM130 in HeLa cells. Cells were co-transfected with pEGFP Mint1 826 and pCDNA3.1 APP 695 RFP and subsequently incubated with anti-GM130 and anti-mouse Alexa488 antibodies. Scale bar: 20 µm. **B)** Co-staining of Mint1 826, APP and GM130 in primary mouse neurons. Cells were co-transfected with pEGFP Mint1 826 and pCDNA3.1 APP 695 RFP and subsequently incubated with anti-GM130 and anti-mouse Alexa488 antibodies. Scale bar: 20 µm. **C)** Live cell imaging of Rab6B and APP. CV1 cells were co-transfected with pDsRed monomer Rab6B Q72L and pEGFP APP 695 and imaged 18 h after transfection.

Further we performed immunocytochemical studies on neuronal cells and non-neuronal cell lines to test for co-localization of Mint1 826 with Rab6 and APP. Analyses of transiently transfected 3T3 Swiss cells with either pEGFP Mint1 or pEGFP Mint1 826 respectively revealed that Mint1 826 is predominantly localized to the Golgi area, whereas Mint1 is distributed evenly in the cytoplasm with no specific accumulation in any cellular compartment (Figure 8A). The quantitative determination of the Mint1 or Mint1 826 level in the Golgi area in comparison to the total amount of the protein inside the cell emphasizes these results (Figure 8B). A similar subcellular distribution of endogenous Mint1/Mint1 826 was observed in primary neurons (Figure 9A). Interestingly, co-staining of EGFP Mint1 826 and Rab6A revealed that both proteins accumulate and co-localize to a high degree in the Golgi area (Figure 8A). Consistently, immunocytochemical analysis of primary mouse neurons transfected with pCDNA3.1 APP696 RFP and pEGFP Mint1 826 revealed that Mint1 826 and APP co-localize with GM130 in HeLa cells as well as in primary mouse neurons at those sites where GFP Mint1 826 accumulates, namely in the Golgi area and in vesicular structures at the periphery of the cells (Figure 9A and B). GM130 like Rab6 is known to localize specifically to the Golgi area [51].This supports our assumption that Mint1 826 is involved in APP sorting in the Golgi and possibly also in the trans-Golgi network and in the secretory pathway. The latter postulation is further supported by live cell imaging studies, showing that Rab6B Q72L and APP clearly co-localize in moving vesicles in the cell periphery (Figure 9 C, Movie S1). Unfortunately, live cell imaging of Mint1 826 could not be carried out due to the toxicity of the overexpressed protein (data not shown).

In conclusion, the demonstration that endogenous Mint1 826 interacts with both APP and Rab6 proteins, as well as the co-localization of APP and Rab6B in moving vesicular structures, support a functional interaction between these proteins. Hereby Mint1 826 might serve as an adaptor protein for the Rab6 regulated transport of APP inside the cell.

## Discussion

In this study, we identified a novel Mint1 variant lacking exon 6, called Mint1 826. We showed that this is a transcribed gene by detection of a specific mRNA sequence and demonstrated the presence of the endogenous protein in tissue samples. This protein is, in contrast to the previously described Mint1 [33], capable of interacting specifically in a nucleotide-dependent manner with the small GTPase Rab6 via its PTB domain. Previous studies in our group demonstrated that the ubiquitously expressed Mint3 is similarly able to bind to GTP-bound Rab6A [18]. Interestingly, in Mint3 the same amino acids are missing in its PTB domain as in Mint1 826, implying that Mint proteins are able to bind to Rab6, when displaying this eleven amino acids deletion.

Our results show that Mint1 and Mint1 826 do not display the same subcellular localization. Mint1 is distributed evenly in the cell, whilst Mint1 826 is highly concentrated in the Golgi area, where it clearly co-localizes with Rab6 (Figure 8). Additional analyses of HeLa cells and primary mouse neurons revealed that Mint1 826 clearly co-localizes with APP 695 and the Golgi marker GM130 (Figure 9A and B). These results suggest an interplay of Mint1 826 and Rab6 in APP sorting. Rab6 also appears to be involved in vesicular APP transport (Figure 9 C), in agreement with previous studies. In 1996 McConlogue and colleagues showed that overexpression of APP and an inactive Rab6A mutant leads to an increased production of soluble APPα by promoting the transport of APP to the plasma membrane [16]. Furthermore, it has been shown that Rab6 promotes the retrograde trafficking of APP from the Golgi apparatus to the ER [17]. On the other hand, more recent studies showing that Rab6 is involved in the transport of exocytotic vesicles towards the plasma membrane via kinesin-1 and the fusion of the vesicles with their target membrane shed new light on the putative role of Rab6 in APP transport [13], [52]. APP is likely to be one cargo of Rab6 that is transported to the plasma membrane, where it is processed in the non-amyloidogenic pathway [25]. Our live cell imaging data support this hypothesis. Rab6B and APP positive vesicles were indeed primarily found in the cell periphery moving towards the plasma membrane (Figure 9 C, Movie S1).

Mint1 826 might be an important adaptor that links Rab6 to its APP cargo. Our studies in primary neurons (Figure 1) support this hypothesis. GST pulldown analyses have shown that Rab6 is not able to bind APP directly (data not shown), suggesting that Mint1 826, which is not only able to bind Rab6, but also APP, could indeed be an adaptor protein for this transport process and therefore might be an important player in the development of Alzheimer’s Disease.

A variety of studies have pointed to the potential neuroprotective effect of Rab6 and Mint proteins by regulating the cellular level of Aβ [53]. The overexpression of Mint3 results in a decrease in the production of Aβ. When the adaptor protein is knocked down, APP transport to the endosomes is increased, which favors processing of APP via the amyloidogenic pathway [53]. It has also been shown that Mint2 controls mechanisms that lead to an accumulation of immature APP in the early secretory pathway therefore suppressing the generation of amyloid beta [54]. Furthermore, studies in mice support the hypothesis that Mint proteins play an important role in the development of AD: AD transgenic mouse models with a Mint1 insufficiency showed an increase in Aβ production [55].

How the different transport processes involving Mint1 826 compare to those regulated by conventional Mint1 has yet to be established. Several groups have already studied the regulation of the Mint adaptor proteins and the impact on APP processing. It has been shown that Mint1 activity is controlled by autoinhibitory mechanisms [56]. In the autoinhibited state the C-terminus of Mint1 binds to the PTB domain and so undergoes a conformational change, which leads to the loss of its APP binding affinity. It is assumed that a phosphorylation of the Tyr633 residue by members of the Src family of non receptor tyrosine-kinases might be the reason for the structural alteration [56]. Indeed, previous studies have shown that Mint proteins are phosphorylated by Src kinases, which influences the intracellular distribution of APP [31]. Future studies will have to clarify, whether these mechanisms apply to the Mint1 826 isoform.

Further investigation on the relation between Rab6, Mint1 826 and APP appears challenging. Although Mint1 knock-out mice are available [38], they most likely show a lack of both the conventional Mint1 and Mint1 826. Also Rab6 knock-out/knock-down experiments do not seem to be appropriate for additional studies, since the different isoforms perform highly overlapping tasks and therefore might be redundant. Nevertheless, additional experiments, comparing the expression levels of Mint1 826 in tissue samples from AD patients, would be informative. Our immunofluorescence analyses showed a partial co-localization between Rab6 and Mint1 (Figure 1). Interestingly, such a co-localization was detected in a restricted number of cells only. The stained protein represents both forms of the adaptor, Mint1 as well as Mint1 826. The fact that rather small areas in a few numbers of cells offer co-localizations with Rab6 indicates that only a low percentage of the cellular Mint1 represents Mint1 826. Whether this ratio is imbalanced in brains of patients suffering from AD has yet to be examined.

The discovery of the new Mint1 826 isoform along with recent results from other research groups including our own group provide new insights into the transport mechanisms involving Rab6, the Mint adaptor proteins and the processing of APP. Yet, further research needs to be performed to establish a more detailed understanding of the complex cellular machineries that are involved in the pathology of AD.

## Supporting Information

Movie S1 Live cell imaging of Rab6B and APP. CV1 cells were co-transfected with pDsRed monomer Rab6B Q72L and pEGFP APP 695 and imaged 18 h after transfection.
(AVI)
